# Utilizing High-Resolution Imaging and Artificial Intelligence for Accurate Leaf Wetness Detection for the Strawberry Advisory System (SAS)

**DOI:** 10.3390/s24154836

**Published:** 2024-07-25

**Authors:** Akash Kumar Kondaparthi, Won Suk Lee, Natalia A. Peres

**Affiliations:** 1 Department of Electrical & Computer Engineering, University of Florida, Gainesville, FL 32611, USA; akash.kondaparth@ufl.edu; 2 Department of Agricultural and Biological Engineering, University of Florida, Rogers Hall, 1741 Museum Road, Gainesville, FL 32611, USA; 3 Department of Plant Pathology, Gulf Coast Research and Education Center, University of Florida, Wimauma, FL 33598, USA; nperes@ufl.edu

**Keywords:** convolutional neural networks, disease management, fungal diseases, image processing, precision agriculture

## Abstract

In strawberry cultivation, precise disease management is crucial for maximizing yields and reducing unnecessary fungicide use. Traditional methods for measuring leaf wetness duration (LWD), a critical factor in assessing the risk of fungal diseases such as botrytis fruit rot and anthracnose, have been reliant on sensors with known limitations in accuracy and reliability and difficulties with calibrating. To overcome these limitations, this study introduced an innovative algorithm for leaf wetness detection systems employing high-resolution imaging and deep learning technologies, including convolutional neural networks (CNNs). Implemented at the University of Florida’s Plant Science Research and Education Unit (PSREU) in Citra, FL, USA, and expanded to three additional locations across Florida, USA, the system captured and analyzed images of a reference plate to accurately determine the wetness and, consequently, the LWD. The comparison of system outputs with manual observations across diverse environmental conditions demonstrated the enhanced accuracy and reliability of the artificial intelligence-driven approach. By integrating this system into the Strawberry Advisory System (SAS), this study provided an efficient solution to improve disease risk assessment and fungicide application strategies, promising significant economic benefits and sustainability advances in strawberry production.

## 1. Introduction

Fungal diseases pose a persistent threat to strawberry crop yield, necessitating effective disease management strategies for growers to maintain economic viability. Anthracnose and botrytis fruit rot, exacerbated by specific environmental conditions, particularly leaf wetness duration (LWD) and temperature, necessitate frequent fungicide applications [1]. Conventionally, growers rely on regular fungicide applications to mitigate disease proliferation, resulting in increased production costs and potential environmental impacts. However, the indiscriminate use of fungicides can lead to pathogen resistance.

Central to the proliferation of these fungal diseases is the presence of free water on plant canopies, measured as leaf wetness duration (LWD), a crucial parameter for assessing disease risk [2]. Accurate measurement of LWD enables growers to make informed decisions regarding fungicide application timing, minimizing unnecessary fungicide use and reducing selection pressure for resistance.

Recognizing these limitations, the Strawberry Advisory System (SAS, http://www.agroclimate.org/sas, accessed on 1 March 2023) was developed to assist Florida’s strawberry growers in making informed decisions about fungicide applications. The SAS is a web-based decision support tool designed to provide guidance on the optimal timing for fungicide applications to manage anthracnose and botrytis fruit rot outbreaks. Data collected by the SAS are available on the AgroClimate website, providing weather monitoring and disease incidence forecasting. The SAS traditionally relies on conductive flat-plate leaf wetness sensors to estimate LWD. However, these sensors are rudimentary and pose challenges, including calibration difficulties, maintenance requirements, and variability in accuracy. While the SAS represents a significant advancement by integrating leaf wetness data with other meteorological conditions, its reliance on conventional sensors underscores the need for more precise and reliable wetness detection methodologies.

Several studies have explored the application of different techniques, such as employing capacitive proximity sensors and various mathematical models to address these challenges, given that prolonged periods of leaf wetness are conducive to the germination and penetration of pathogenic fungi into plant tissues [3,4]. Infrared cameras have also been employed to detect water droplets based on temperature changes. Utilizing infrared thermography, thermal imaging techniques detect the cooling effect of evaporation from wet surfaces. This method is beneficial in large-scale monitoring, providing a non-invasive way to detect leaf wetness across extensive agricultural fields [5]. Traditional methods for measuring leaf wetness, such as mechanical and electronic sensors, have been pivotal in monitoring this crucial parameter [6].

Recent studies have explored the feasibility of using imaging-based devices and lasers for leaf wetness detection. Internet of Things (IoT) sensors and computer vision techniques for environmental monitoring represent a significant leap forward in precision agriculture, enabling more detailed and accurate analyses of crop conditions [7,8,9]. Laser reflection was used to measure leaf wetness utilizing the fact that the presence of water changed the reflected laser signal from the leaf surface, but the testing was conducted only under a controlled environment [10]. Leaf wetness was also measured by illuminating a red laser on a leaf surface as water presence on leaves reduces the red channel intensity, but this study was also conducted only in a laboratory environment [11]. Another study demonstrated measuring water molecules on a leaf surface using graphene oxide as a sensing film, but its response time was slow (400 s) [12]. A geostationary satellite was also used to estimate leaf wetness duration with machine learning algorithms [13].

Deep learning, a subset of AI focusing on neural networks with multiple layers, has demonstrated remarkable success in image classification tasks, surpassing traditional machine learning methods in both accuracy and efficiency [14,15]. Convolutional neural networks (CNNs), in particular, have been effectively applied in various agricultural applications, including disease detection, plant species identification, and monitoring environmental conditions [16,17,18]. The enhancement of such systems through the incorporation of AI-based leaf wetness detection methods represents a crucial step toward more accurate and reliable disease management strategies [19].

Initial implementations of a leaf wetness detection system at the University of Florida’s Plant Science Research and Education Unit (PSREU) in Citra, Florida, USA, have shown promising results, prompting the expansion of the project. The project was implemented earlier in 2021 and since has been expanded to three other locations across Florida: Florida AG Research, Dover; the University of Florida’s Gulf Coast Research and Education Center (GCREC), Wimauma; and Fancy Farms, Plant City.

The objective of this study was to improve the in-field imaging-based leaf wetness detection systems (named Imaging System hereafter) and evaluate their performance by exploring the use of convolutional neural networks (CNNs). The camera quality of the system in Plant City was upgraded to test for improvement in model performance. The algorithm developed then divided the regularly acquired images into different categories based on the time of the day of the image taken and the respective image quality. Since different lighting conditions and weather conditions created different images, different models were trained to classify different sets of data. For example, images with water droplets due to rain on the camera lens were classified into a class different from images with dim lighting conditions due to cloudy weather. Through field trials conducted at four separate locations during the strawberry growing season, the performance of the developed system was assessed against manual observations and SAS data.

## 2. Materials and Methods

A wetness detection system previously developed and placed at the Plant Science Research and Education Unit (PSREU) of the University of Florida (UF) in Citra, Florida, USA, used a relatively lower resolution camera (Wyze Web camera v2, Wyze Labs, Seattle, WA, USA). The same system was also set up at two other locations: the Gulf Coast Research and Education Center (GCREC) of the University of Florida, Wimauma, Florida, USA, and Florida AG Research, Dover, FL, USA. The systems at these locations collected data from May 2023 to April 2024. All these systems were placed adjacent to the strawberry fields no farther than 10 m from the fields. In October 2023, a new system with a new camera (details below) was developed and installed at a commercial strawberry field (Fancy Farms) in Plant City, FL, USA, the data obtained from which were used in this study.

**Figure 1:**
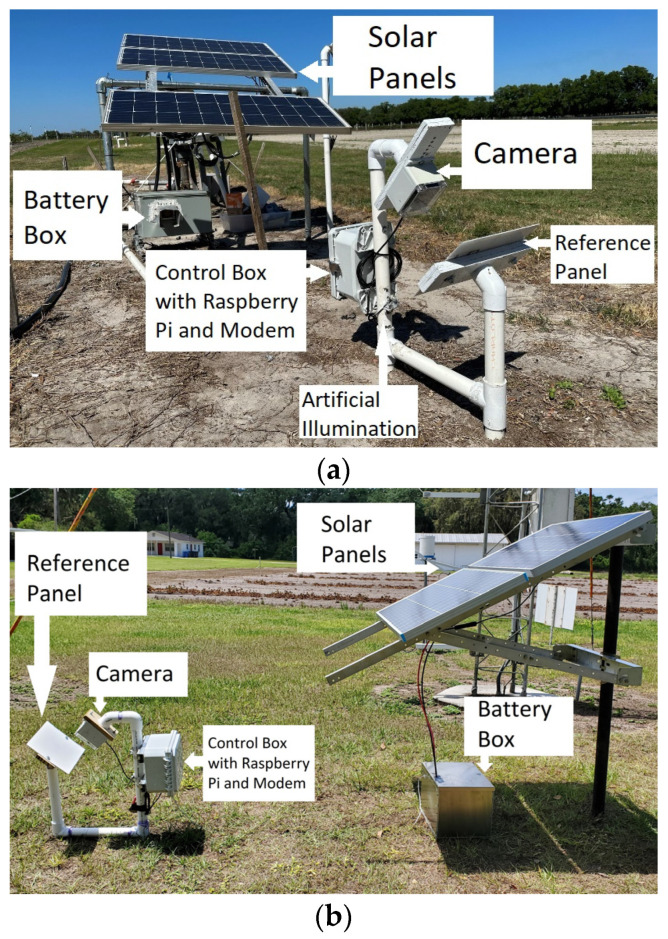

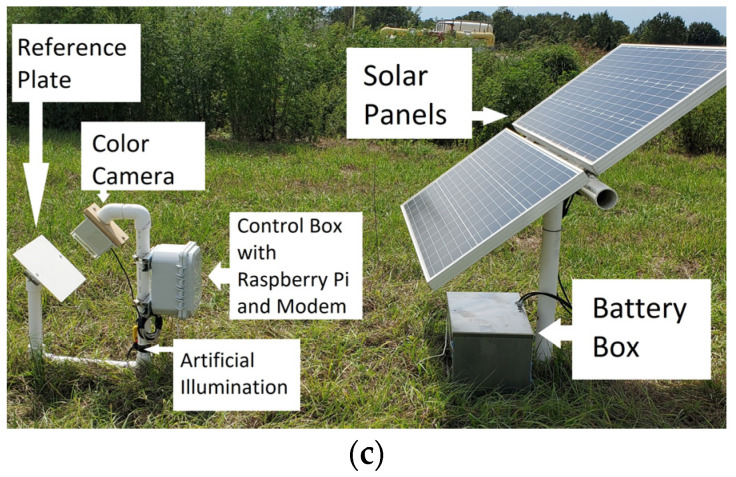
Wetness detection system at (**a**) UF PSREU, Citra, (**b**) Florida AG Research, Dover, and (**c**) UF GCREC, Wimauma.

**Figure 2:**
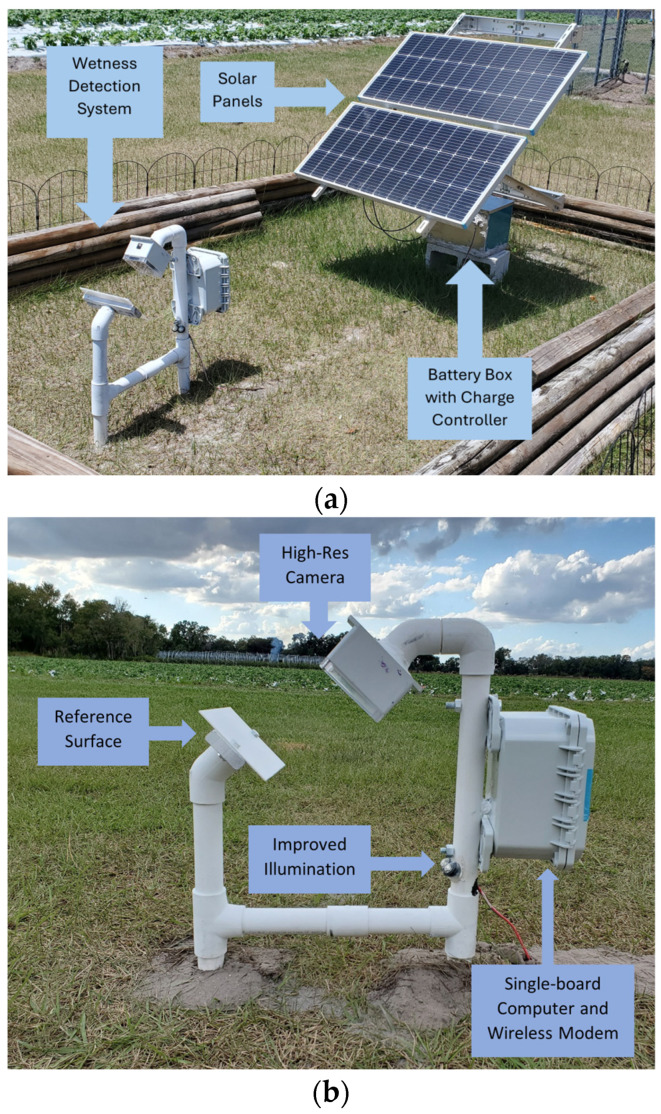
New system setup at Fancy Farms, Plant City: (**a**) whole system view, and (**b**) wetness detection system components at Plant City. A high-resolution camera and more powerful LEDs were implemented in this system, compared to the previous ones in Figure 1.

Figure 1a shows the system setup at PSREU in Citra, and Figure 1b,c show similar systems placed at Dover and GCREC, respectively. Figure 2a,b show the new system placed at Fancy Farms, Plant City.

### 2.1. System Design and Setup

The new system setup used the following components:

- *A High-Resolution Camera:* To improve the resolution of the images to facilitate the detection of droplets of smaller sizes, a switch to a high-definition camera was made. A Raspberry Pi Camera Module 3 (Raspberry Pi Foundation, Cambridge, UK) was used in the new system that took images at a resolution of 4608 × 2592 pixels, whereas the previous camera (Wyze Web camera v2, Wyze Labs, Seattle, WA, USA) only took images of resolution 1920 × 720 pixels. This huge improvement in the resolution enabled a better observation of smaller water droplets on the reference plate.
- *Reference Plate:* A flat white non-reflective reference plate was used. The reference plate is made of an acrylic sheet (Duraplex Clear Acrylic Sheet, Duraplex, Brick, NJ, USA) and is painted with a non-reflective flat white paint (Flat White General Purpose Spray Paint, Rust-Oleum, Vernon Hills, IL, USA). The plate was made sure to be flat without bumps so as not to be confused by the model with a water droplet. The previous plate size was 20.3 cm × 25.4 cm, but the size was reduced to 12.7 cm × 19 cm to bring the panel dimensions closer to the commercial wetness sensor dimensions.
- *LED Lighting:* To accommodate the night-time image capturing, two white LED lights were used for night-time illumination. These lights were controlled by the computing unit so that they were turned on only during the image capturing instances to save power and avoid attracting insects. The LEDs were placed such that the light fell on the plate at approximately a 10-degree angle to extenuate the droplet shadows for better detection.
- *Computing Unit:* A single-board computer (Raspberry Pi 3, Raspberry Pi Foundation, Cambridge, UK) was used as the processing unit for the system. It took care of periodically acquiring the images using the camera, turning the LEDs on during the image capturing process, saving the files, cropping the images, uploading the images to Google Drive through a connected wireless modem (Verizon Jetpack MiFi 8800L, Verizon Communications Inc., New York City, NY, USA), and running the wetness detection algorithm through the obtained images.
- *Solar Panel Setup:* Two solar panels (Renogy 100 Watt 12 Vol Monocrystalline Solar Panel, Renogy, Thailand) of 100 W power each with a battery (Deep Cycle AGM Battery 12 Volt 100Ah, Renogy, Thailand) of 100 Ah were used with a charge controller (Wanderer Li 30A PWM Charge Controller, Renogy, Thailand) to provide the power to the system.

### 2.2. Data Collection and Model Training

**Figure 3:**
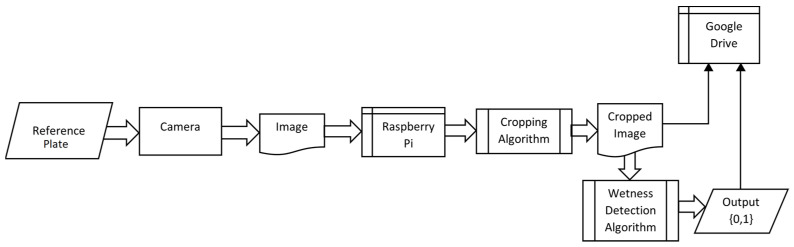
System block diagram.

As shown in Figure 3, the camera took an image of the reference plate every 15 min. Each image was taken at the full resolution of the camera: 4608 × 2592 pixels. The original images were stored separately, and the cropping algorithm cropped the images to the center to only include the reference plate and exclude the background, since the droplets could only be observed on the reference plate. The images were cropped to the center at a resolution of 2100 × 1600 pixels.

The data were collected from 30 October 2023 to 29 March 2024, comprising 9429 images. These images were divided into a 90% training set (including the validation set) with 8485 images and a 10% test set with 944 images.

### 2.3. Image Processing and Algorithm Development

The current benchmark stands at 94.4% accuracy when compared to manual observations [16]. The drawbacks of the previous model consisted of detecting smaller droplets and detection during ambient conditions which were worsened by the image quality of the older low-resolution camera. In this study, the goal was to surpass the current benchmark accuracy obtained by the previous model, when compared to manual observations and to the wetness data from the Strawberry Advisory System (SAS). The labels for the manual observations were assigned by manually looking at every image in the collected dataset and subsequently assigning the respective label based on the presence of water droplets in the image.

The Strawberry Advisory System (SAS) employs a sophisticated data collection strategy to optimize the timing of fungicide applications. The SAS utilizes Campbell Scientific model 237 sensors, placed in various locations within the fields. To enhance the reliability of the wetness data, the SAS integrates outputs from four distinct leaf wetness models. This multi-model system helps resolve discrepancies when sensor readings do not align, ensuring a more accurate assessment of the leaf’s wetness state. Each model processes data independently, and a consensus approach determines the final wetness status based on the majority agreement among the models. The system further refines the data through algorithmic processing, which assesses the combined sensor and model outputs. This process ensures that the advisory system provides the most accurate and timely recommendations for fungicide application.

The proposed method in the current study was to use a higher-resolution camera to enable the observation of smaller droplets and detection during ambient conditions with better quality images and to use multiple models for detection instead of using a single model for the entire dataset.

**Figure 4:**
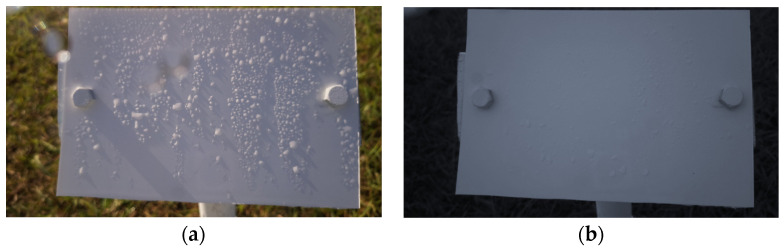
(**a**) A regular (Day class) image and (**b**) a Blue class image.

The proposed wetness detection algorithm consisted mainly of two steps, Time-of-Day classification and wetness classification, which are explained below. For the following section, the image shown in Figure 4a will be considered a “regular image instance” with usual sunny lighting conditions for comparison.

**Figure 5:**
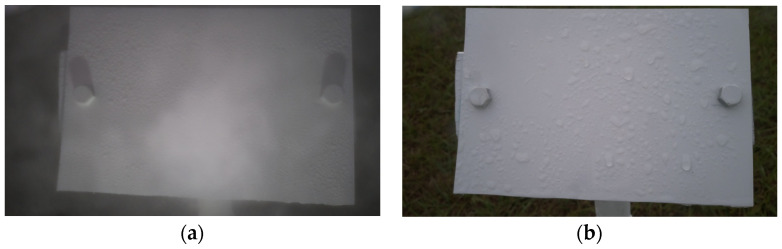
(**a**) A Blurry image and (**b**) a Cloudy image.

**Figure 6:**
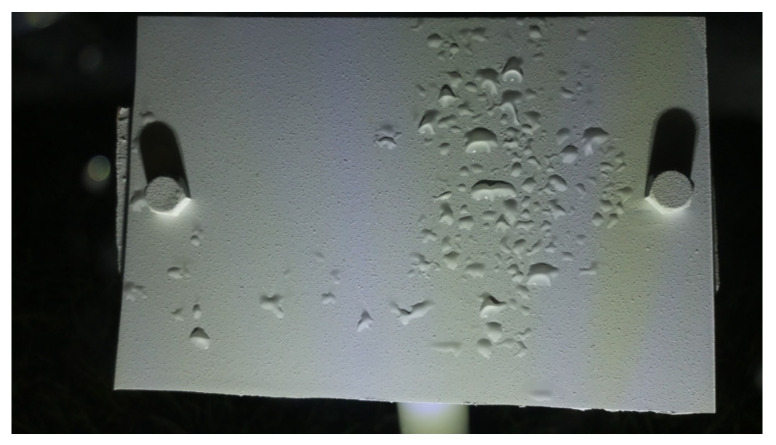
An image taken during the Night.

- Time-of-Day Classification: The entire dataset consisted of images of a wide variety of illumination conditions, droplet sizes, brightness levels, clarity levels, and weather conditions. The droplet appearances in all these classes varied greatly. Using a single model to learn the features in all the conditions might not be the optimal solution. Hence, this proposed algorithm first classified the images into the following five categories:
  *(1)*  *Blue:* These images were taken during dawn or at dusk and had some blueish tint over the image. Since these images had different compositions of the intensity values over the three channels, the features in these instances were different from a regular image. The visibility of the droplets was not optimal since the droplet shadows were not entirely visible. This class required a more complex model to preprocess the images to bring up the model visibility. Figure 4b shows an example image of a Blue class image.
  *(2)*  *Blurry:* There were some instances in the dataset where when it rained a lot; the water droplets also covered the camera lens and made the entire image blurry as a result. These images were relatively easier to classify since they stood out from a regular image. These images can directly be classified as Wet since the droplets are what makes an image class Blurry. Figure 5a shows an instance where the lens was covered with water droplets making the entire image blurry.
  *(3)*  *Cloudy:* These images were the ones that were taken during cloudy weather conditions. These conditions made the image tone look more grayish. As a result of low brightness and lack of a uni-directional light source, these images had ambient lighting conditions where the droplet visibility was also reduced. Similar to the Blue class images, these images needed a model of a relatively higher complexity. Figure 5b shows an example of a Cloudy image.
  *(4)*  *Day:* A Day class image (Figure 4a) was one taken on a sunny day with no clouds obscuring the sun. This is what was established as a regular image. The sunlight made the droplet visibility good and all parts of the image were distinguishable.
  *(5)*  *Night:* These images were taken at night, when there is no light from the sun. Night class images were solely illuminated by the artificial illumination source, the LEDs. Since these LEDs were already optimally placed to maximize the droplet shadows, this class of images is the easiest, in theory, to detect the droplets. Figure 6 shows a Night class image.

**Figure 7:**
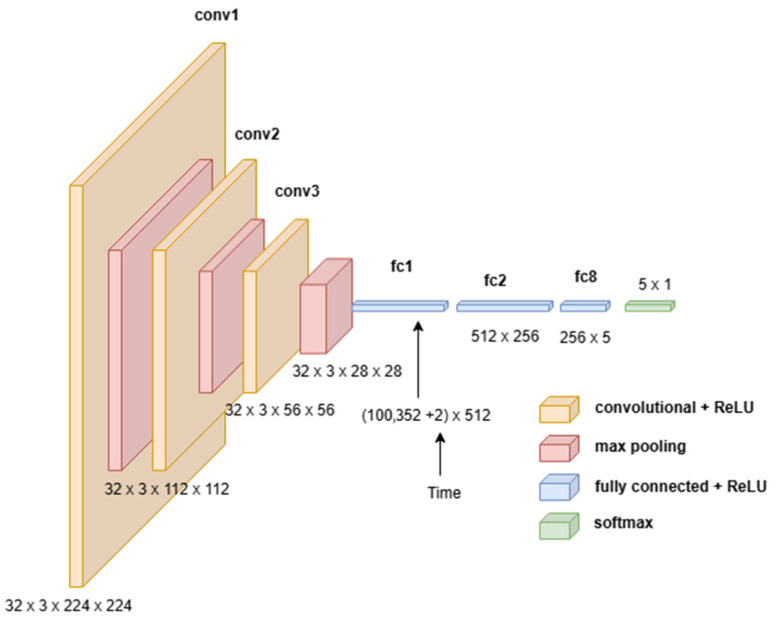
The architecture of the convolutional neural network layers used for the Time-of-Day classification.

**Figure 8:**
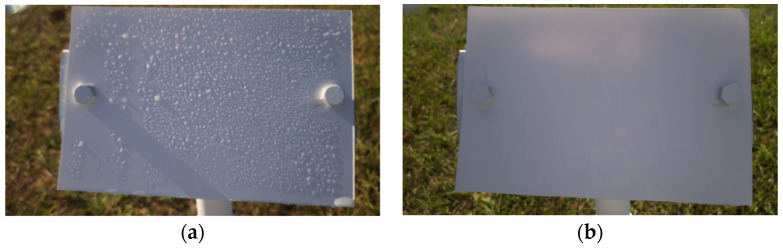
Example images of the reference plate during the Day: (**a**) Wet image and (**b**) Dry image.

**Figure 9:**
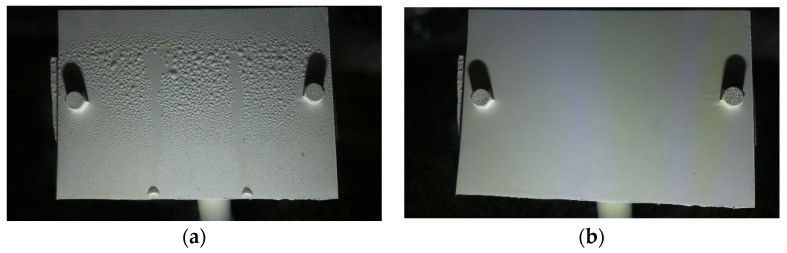
Example images of the reference plate at Night: (**a**) Wet image and (**b**) Dry image.

This Time-of-Day classification took uncropped images, since the image background also has a lot of information regarding the illumination conditions. The model trained on this classification was also provided with the time when the image was taken to better classify the images into the five categories. When an image was taken, the timestamp of the exact instant was also stored with the image itself. When the image data were provided to the model for training and testing, the timestamp data of the specific image were also provided. The timestamp was in 24-h format stored with year, month, date, hour, minutes, and seconds. Only the two values, hour and minutes, were provided to the model, as shown in Figure 7, where the time value accounted for an additional two parameters (hour and minutes). Furthermore, since the visual signature of the time of the image taken could be observed in the lighting conditions and the detail in the image did not matter, the images for this classification were resized down to 224 × 224 pixels from the original images size of 4608 × 2592 pixels, making sure that the visual signature was not lost. The model architecture used for training on these Time-of-Day classifications is illustrated in Figure 7. The training set for this model training included 9429 images and the test set included 944 images. The time element was introduced in the first dense layer by concatenating the time information to the flattened output from the last convolutional layer. This enabled the model to consider the time of the image and make the classification more sensible.

b. Wetness Classification: After classifying the images into Time-of-Day categories, these images of different classes were independently trained using CNN-based wetness detectors. A training set of 2784 images, a validation set of 280 images, and a test set of 310 images were used for this classification. Since the Blurry images already had droplets in them, they were directly classified as Wet. Due to the observed high correlation between the Blue and Cloudy images, a single model was trained on the combined dataset of Blue and Cloudy images for wetness classification. Figure 8 shows typical examples of Wet and Dry images during the Day. Because of the presence of brighter and stronger sunlight, the visibility of the water droplets is also increased. Figure 9 shows typical Wet and Dry images at Night. In the Night images, the droplet visibility is exaggerated due to the angle of the illumination.

**Figure 10:**
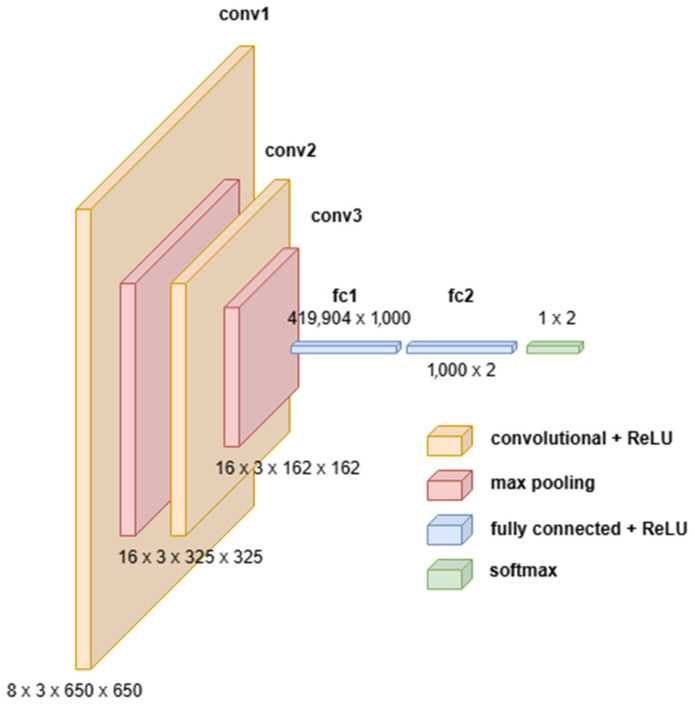
The architecture of the model layers used for the wetness classification.

A wetness detector CNN model architecture used for the wetness classification of the images is shown in Figure 10. As can be observed from the illustration, it is apparent that the model is much simpler and computationally lighter compared to the previous model by Patel et al. (2022) [16] or the Time-of-Day classifier.

Table 1 shows the number of images used in the training, validation, and test sets for the Time-of-Day classification and all the wetness classifications. The Time-of-Day dataset included images from all the classes, whereas the wetness classification datasets included only the datasets of the respective classes.

### 2.4. Evaluation Methods

Several evaluation metrics can be used to assess the performance of these models. However, since this task is a classification task, the accuracy score proved to be the best metric. The accuracy score can be calculated as follows:

$$Accuracy=\frac{TP+TN}{TP+TN+FP+FN}$$

where TP = True Positives, TN = True negatives, FP = False Positives, and FN = False Negatives.

The results from the obtained models were compared to manual labels, the data obtained from the Strawberry Advisory System (SAS), which uses a combination of two leaf wetness sensors and LW models [20], and the results from the previous model. The comparisons were carried out by calculating correlations, accuracy scores, precision scores, recall scores, and plotting confusion matrices.

## 3. Results

**Figure 11:**
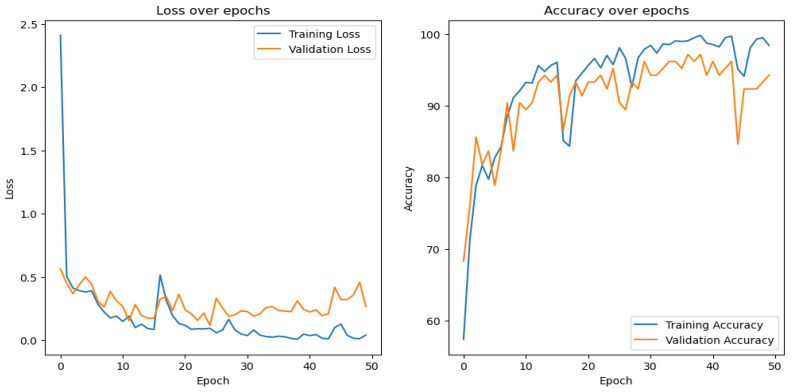
Learning curves of the Time-of-Day classification model.

The models were trained to classify the images with manual labels. Figure 11 shows the learning curve obtained from training the model shown in Figure 7 on the Time-of-Day classification dataset. It is apparent from the learning curves that the accuracy increases in an expected fashion and the loss decreases normally. The training was stopped after just a few epochs when the model started to overfit. The stochasticity in the curves is a result of training the model with a smaller batch size.

**Figure 12:**
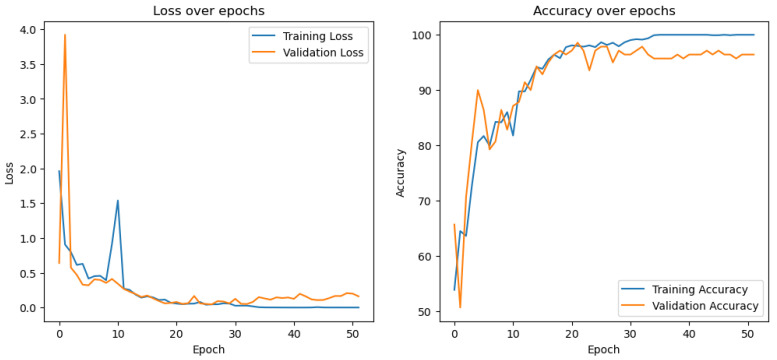
Learning curves of the wetness classification model.

Figure 12 shows the learning curves obtained from training a model, as shown in Figure 10, on the wetness classification dataset. The accuracy also increases, and the loss decreases normally right before it starts to overfit. In this case too, the training was stopped after just a few epochs when the model started to overfit. Early stopping was implemented so that only the best model with the best (least) validation loss in the epochs was saved for testing.

### 3.1. Results from the Time-of-Day Classification

The model trained on the Time-of-Day classification outputted the following results: the training accuracy was 95.3%, the validation accuracy was 92.9%, and the test accuracy was 92.0%.

**Figure 13:**
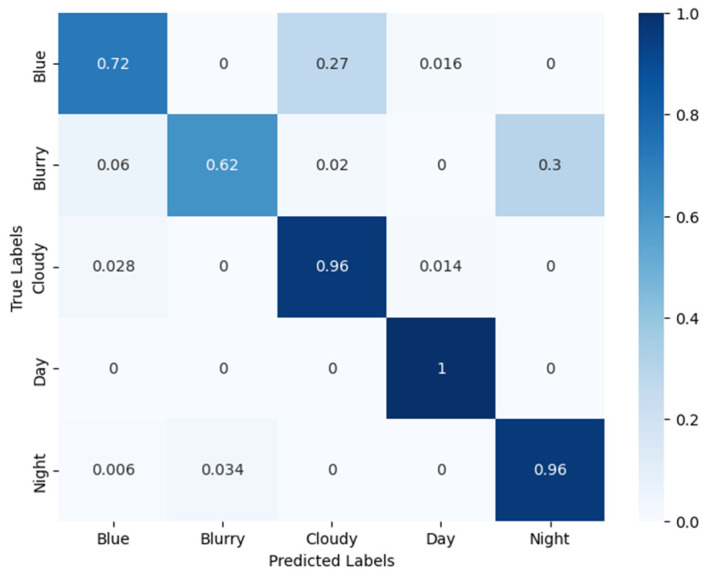
Confusion matrix of the Time-of-Day classification.

A more in-depth analysis of these results can be inferred from the confusion matrix. Figure 13 shows the normalized confusion matrix of the Time-of-Day classification results on the test set. The diagonal numbers in the matrix represent the percentage of the total images that were correctly classified. The model excels at classifying Cloudy, Day, and Night class images, which is apparent from the higher percentages in the diagonal entries corresponding to the Cloudy, Day, and Night classes. However, the model needs improvement in the Blue and Blurry classes, since these diagonal entries have fewer percentages representing the images correctly classified. From the matrix, it is also apparent that the Blue class images were also misclassified as Cloudy images. This was because the Blue class images were closely correlated with the Cloudy class images, for which the reason found was the low-intensity levels in the Blue and Cloudy classes. Hence, a single model was used to classify the Blue and Cloudy images into Wet or Dry instances in the wetness classification stage.

### 3.2. Results from the Wetness Classification

A model was trained on the combined dataset of Blue and Cloudy class images due to their close resemblance. The accuracy results are as follows when compared to the manual labels. The training accuracy was 98.2%, the validation accuracy was 97.9%, and the test accuracy was 97.4%.

**Figure 14:**
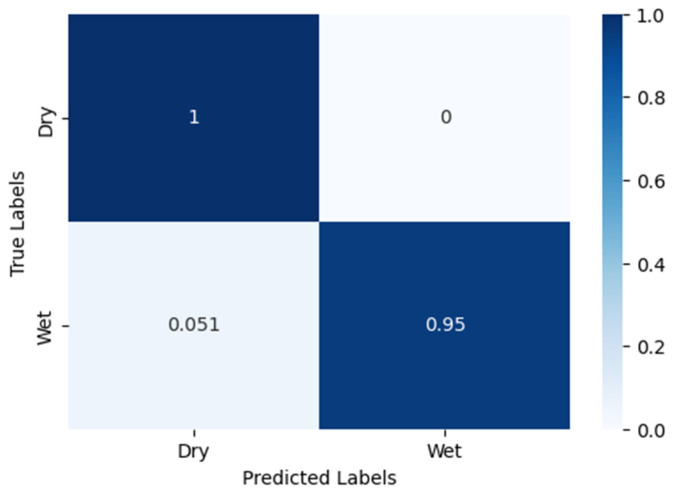
Blue–Cloudy classification confusion matrix.

From the confusion matrix of these results shown in Figure 14, the model perfectly isolated the Dry class images, although there was a small error in classifying Wet images. This makes sense because since the lighting conditions of these images were relatively poor, the droplet visibility was largely reduced, which made the model mistake a Wet instance as a Dry instance.

Another model was trained on the dataset of Day class images, whose accuracy results are as given when compared to the manual labels. On a training set of 2565 images, the training accuracy was 99.6%. On a validation set of 256 images, the validation accuracy was 96.5%, and on a test set of 385 images, the test accuracy was 98.9%.

**Figure 15:**
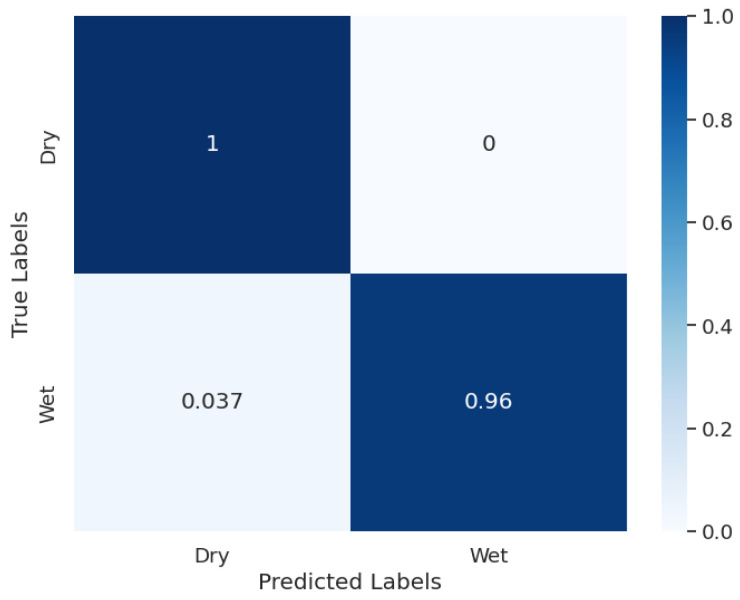
Day classification confusion matrix.

The normalized confusion matrix of Day classification results is shown in Figure 15. Similar to the Blue–Cloudy model, the Day classification model also perfectly isolated the Dry class images, although there was a small error in classifying Wet images. The reason stays the same since during the very early stages of the dawn, the angle of the sun was so low that the reference plate was not fully illuminated, making the visibility of droplets difficult.

Finally, a different model was developed and trained on the dataset of Night class images. Since these images were the easiest of the whole dataset, a model with relatively lower complexity was used with a smaller number of convolutional kernels. The accuracy results are as provided when compared to the manual labels. On a training set of 3285 images, the training accuracy was 98.8%. On a validation set of 296 images, the validation accuracy was 97.5%, and on a test set of 385 images, the test accuracy was 97.3%.

**Figure 16:**
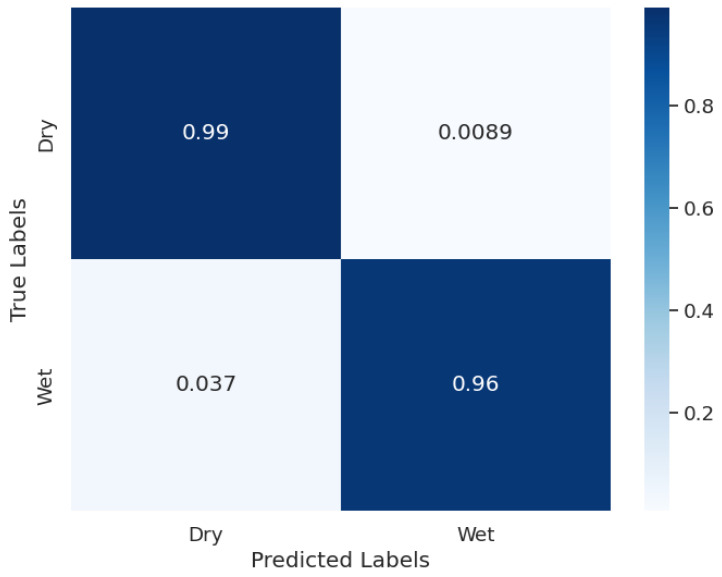
Night classification confusion matrix.

The normalized confusion matrix of Night classification results is shown in Figure 16. Unlike the previous models, the Night classification model had both False Positives and False Negatives. The main reason for the False Positives (Wet classified as Dry) was that the instances of dew onsets had very small droplets and the model could not detect them. The False Negatives (Dry classified as Wet) happened when dust particles on the plate were falsely detected as water droplets.

### 3.3. Overall Algorithm Results

**Figure 17:**
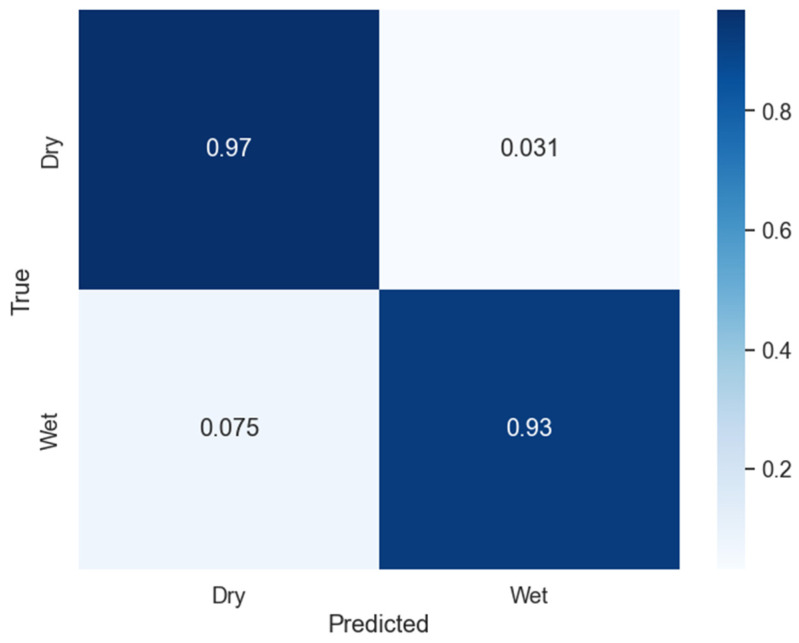
Confusion matrix of the overall algorithm.

Combining all the trained models into one algorithm, the overall result was calculated on a test set of 8896 images as shown in Table 2. The overall accuracy for the manual labels was 95.8%, and when compared to SAS data, it was 83.8%. The confusion matrix of the entire algorithm is shown in Figure 17.

## 4. Discussion

From the results presented in the last section, it can be concluded that there is a significant improvement in the model performance compared to the one presented by Patel et al. (2022) [16]. The new algorithm performed well, resulting in a correlation of 95.75% when compared to manual observations. This correlation dropped to 83.78% when compared to the SAS data, although the correlation between SAS data and the manual observations was 90.30%.

**Figure 18:**
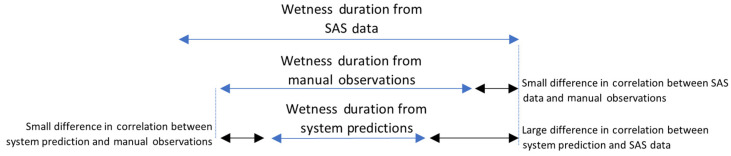
Illustration of the differences in correlations of wetness durations (LWDs).

Upon careful inspection of the data from all three sources, the LWD (or wetness durations) in the Imaging System predictions and the manual observations was lower than that from the SAS data. The wetness durations were shorter in the Imaging System predictions, meaning the system was detecting wetness with a higher threshold, as illustrated in Figure 18. The manual observations had wetness for slightly longer durations, and the SAS data had the longest, meaning that the SAS data were detecting wetness with the lowest threshold. The reason for the highest correlation between manual observations and Imaging System predictions, followed by the correlation between manual and SAS data, and the least correlation between SAS and system predictions can be inferred from Figure 18.

**Figure 19:**
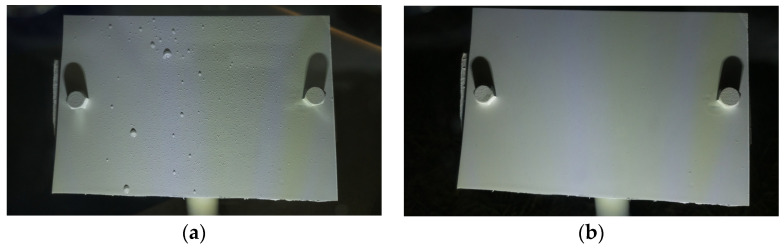
Correctly classified instances at Night: (**a**) Wet image and (**b**) Dry image.

**Figure 20:**
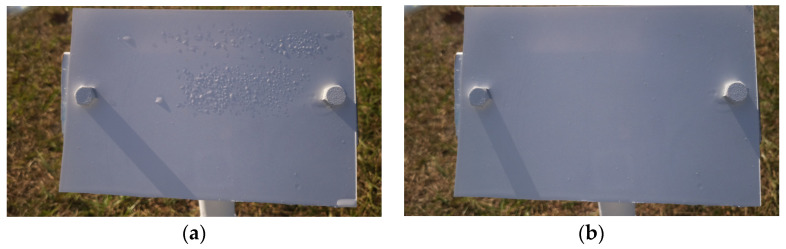
Correctly classified instances during the Day: (**a**) Wet image and (**b**) Dry image.

Looking at the strengths of the algorithm, images where the droplets were clearly visible had no difficulty in being classified into Dry or Wet. Figure 19 shows example images of a Dry instance and a Wet instance from the Night class where the new algorithm succeeded in correctly classifying them into Wet or Dry classes. Figure 20 shows example images of a Wet instance and a Dry instance from the Day class where the new algorithm succeeded in the correct classification of images.

**Figure 21:**
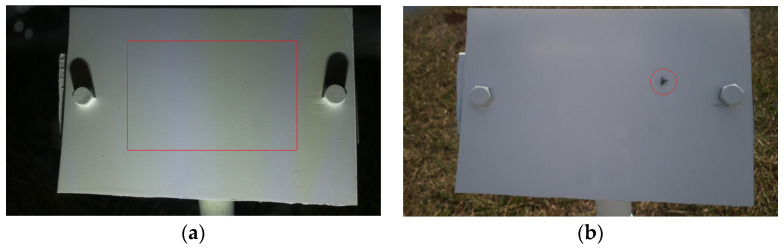
False positives due to: (**a**) dust particles all over the image and (**b**) insects on the reference plate indicated by the red circle.

The overall algorithm has a small error in this classification, as can be observed from Figure 17. From the confusion matrix, 3.1% of False Positives can be observed. False Positives are the instances that are actually Dry but classified by the algorithm as Wet. Figure 21 shows a few examples of the False Positives. Occasionally, dust particles accumulated on the reference plate and contributed to the wetness detection error. This remained a problem for the current system, as shown by the red box in Figure 21a. Some insects flying over the reference plate also contributed to this error, as shown by the red circle in Figure 21b.

**Figure 22:**
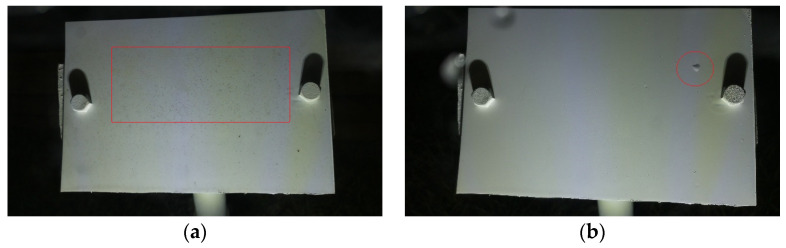
False Negatives due to: (**a**) dew onset with very tiny droplets and (**b**) lower number of droplets.

The False Negatives were the ones that were indeed Wet but were classified as Dry by the algorithm. False Negatives contributed 7.5% to the total error. For these instances, the system predicted Dry instead of Wet because, most of the time, the water droplets were not so clearly visible due to their very tiny size (less than 1 mm). This was mostly during the onset of dew formation, where the droplet size was very small, as shown in Figure 22a, inside the red frame. Figure 22b shows an example of an instance where the droplets were lower in number. As in the case of Figure 22b, only one droplet can be observed encircled by a red circle.

Even with the improved performance, the new system still has some room for improvement. During this research, some issues were encountered such as color degradation of the reference plate, power outages, and network connectivity issues. The most important problem with this setup is that the reference plate will degrade after roughly five months and will need to be repainted or replaced. This happens due to various factors such as dust, overexposure to the sun, and bird droppings, and manual maintenance is needed for the system to keep running for longer than half a year. These reasons also contributed to the False Positives in the results. In the future, these problems can be overcome by implementing wipers, or different materials for the reference plate, bypassing the need for manual maintenance. Furthermore, the challenge of detecting very tiny droplets during the onset of dew can be tackled with the application of time series models, which can capture the phenomenon of growing droplets [21].

## 5. Conclusions

This research presents a significant advancement in precision agriculture with the development of a highly accurate leaf wetness detection system using imaging and deep learning. By employing convolutional neural networks to analyze high-resolution images of a reference plate, this study introduces an innovative divide-and-conquer approach that classifies images based on different times of the day and weather conditions. The introduction of high-definition cameras enabled the detection of smaller water droplets, enhancing the system’s sensitivity and precision. This method has been shown to enhance the reliability of leaf wetness measurements, which are critical for managing fungal diseases in many crops.

The system achieved an overall accuracy of 95.75%, demonstrating its efficacy in detecting leaf wetness with high precision. The implementation of this advanced detection system within the SAS may improve decisions about fungicide applications and has the potential to be expanded to other disease support systems. Further improvement could focus on the detection of smaller droplets during the onset of dew formation, possibly by integrating time series predictive models to track and predict changes in wetness over time.

## Figures and Tables

**Table 1:** Dataset sizes used for the Time-of-Day and wetness classifications.

	Time-of-Day	Blue and Cloudy	Day	Night
Training Set Size	7637	2784	2565	3285
Validation Set Size	848	280	256	296
Test Set Size	944	310	385	385
Total	9429	3374	3206	3966

**Table 2:** Accuracy scores of the overall algorithm.

Comparison	Accuracy Scores
Number of images	8896
Manual observation vs. Image detection system	95.8%
Manual observation vs. SAS	90.3%
Image detection system vs. SAS	83.8%

## Data Availability

The datasets presented in this article are not readily available because the data are part of an ongoing study.
